# Deep learning techniques for imaging diagnosis and treatment of aortic aneurysm

**DOI:** 10.3389/fcvm.2024.1354517

**Published:** 2024-02-28

**Authors:** Legang Huang, Jiankuan Lu, Ying Xiao, Xiaofei Zhang, Cong Li, Guangchao Yang, Xiangfei Jiao, Zijie Wang

**Affiliations:** Department of Vascular Intervention, Shengli Oilfield Central Hospital, Dongying, China

**Keywords:** deep learning, artificial intelligence, aortic aneurysm, prediction model, imaging diagnosis

## Abstract

**Objective:**

This study aims to review the application of deep learning techniques in the imaging diagnosis and treatment of aortic aneurysm (AA), focusing on screening, diagnosis, lesion segmentation, surgical assistance, and prognosis prediction.

**Methods:**

A comprehensive literature review was conducted, analyzing studies that utilized deep learning models such as Convolutional Neural Networks (CNNs) in various aspects of AA management. The review covered applications in screening, segmentation, surgical planning, and prognosis prediction, with a focus on how these models improve diagnosis and treatment outcomes.

**Results:**

Deep learning models demonstrated significant advancements in AA management. For screening and diagnosis, models like ResNet achieved high accuracy in identifying AA in non-contrast CT scans. In segmentation, techniques like U-Net provided precise measurements of aneurysm size and volume, crucial for surgical planning. Deep learning also assisted in surgical procedures by accurately predicting stent placement and postoperative complications. Furthermore, models were able to predict AA progression and patient prognosis with high accuracy.

**Conclusion:**

Deep learning technologies show remarkable potential in enhancing the diagnosis, treatment, and management of AA. These advancements could lead to more accurate and personalized patient care, improving outcomes in AA management.

## 1 Introduction

Aortic Aneurysm (AA) refers to the permanent dilation of the human aorta, with a diameter exceeding 1.5 times that of the normal aorta, often resulting from atherosclerosis and hypertension. The occurrence of AA is associated with various epidemiological factors, such as age, gender, race, family history, and smoking (1). AA is a life-threatening condition, and its treatment depends on surgical repair, which can be achieved through open surgery or endovascular aneurysm repair (EVAR). The guidelines from the European Society for Vascular Surgery offer recommendations for the management of patients with AA, suggesting that the treatment plan should be based on balancing the assessed risks of surgery with the risks of growth and rupture of the AA. Computed tomography angiography (CTA) imaging remains the most commonly used technique in surgical planning, as it provides a comprehensive dataset of the entire aorta and access vessels, allowing for the assessment of the extent and morphology of the AA and the identification of coexisting occlusive diseases (2, 3).

Deep learning, often referred to as a subset of artificial intelligence (AI), is an important branch of machine learning technology. Compared with traditional machine learning techniques such as Support Vector Machines (SVM), Random Forests, Decision Trees, K-Nearest Neighbors (KNN), Naive Bayes, and Logistic Regression, deep learning employs different training models and methods. Traditional machine learning relies on manually extracted features and clearly defined algorithmic rules, whereas deep learning models, especially Convolutional Neural Networks (CNN), significantly enhance the accuracy of machine learning by automatically learning complex feature representations from vast amounts of data. With the continuous iteration and improvement of model complexity, machine recognition capabilities have reached human-level performance for the first time (4), driving significant developments in the field of artificial intelligence and reshaping various aspects of production and daily life, including applications such as AlphaGo, facial recognition for payments, and autonomous driving.

Different deep learning models, including CNN, Recurrent Neural Networks (RNN), Bayesian Neural Networks (BNN), and Graph Convolutional Networks (GCN), each have their unique structures and training methods, suitable for different types of data and tasks. For example, CNNs excel in image processing and visual recognition tasks, while RNNs are more suitable for sequential data such as text and speech. BNN introduce probability distributions into network parameters, offering a method to handle uncertainty and assess prediction credibility, which is particularly important in areas requiring highly reliable predictions like medical diagnosis and financial analysis. GCN extend deep learning to graph-structured data, enabling the network to learn directly between nodes in a graph, applicable to tasks such as social network analysis and protein structure prediction. Unlike traditional predictive models, such as regression models that directly establish mathematical relationships between inputs and outputs, deep learning models learn abstract representations of data through multiple layers of nonlinear transformations, enabling them to capture more complex patterns and relationships. The diversity and flexibility of these models are key to the success of deep learning across various fields.

**Figure 1:**
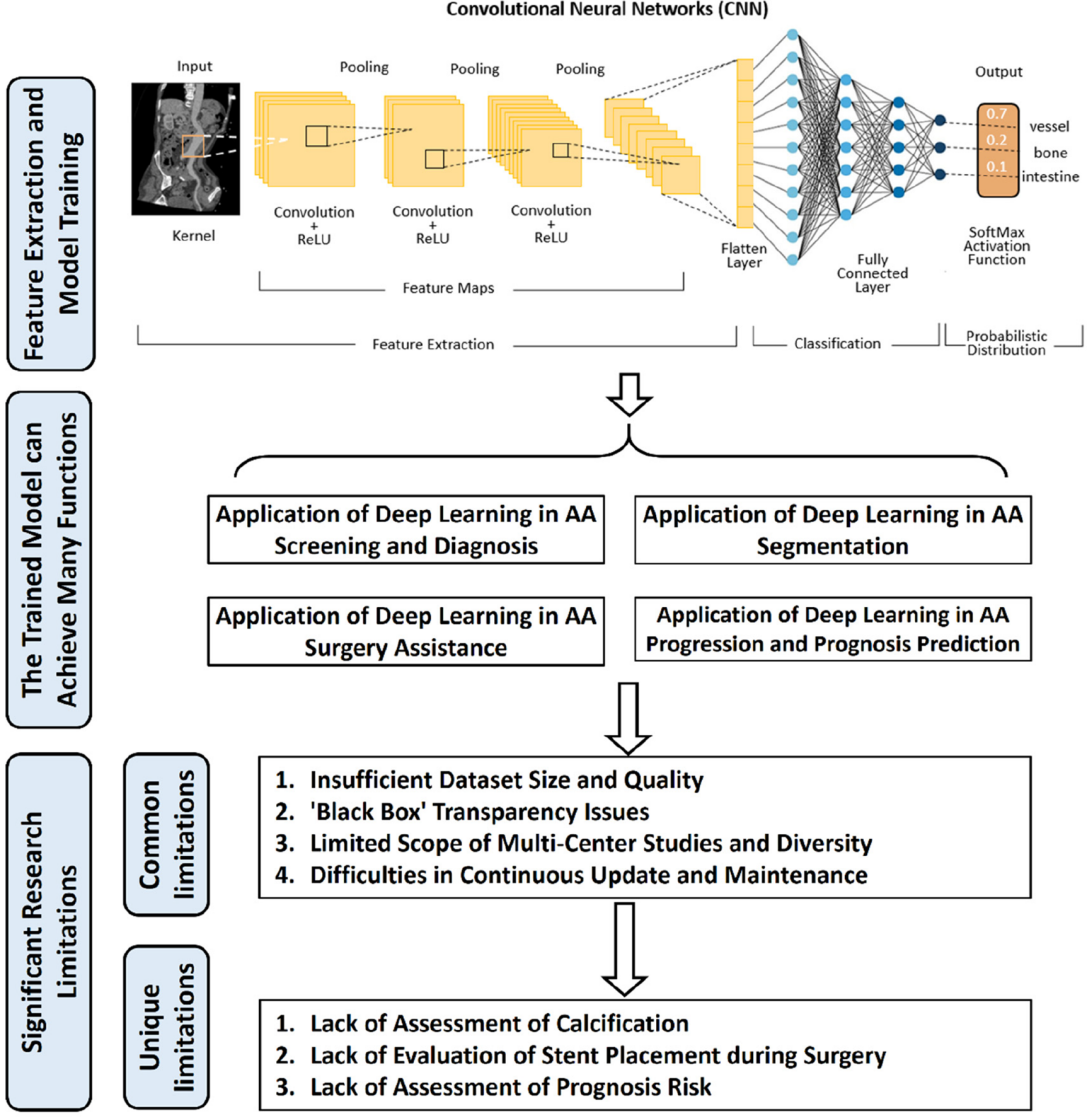
The workflow and application of deep learning research in radiological image data from patients with aortic aneurysms (AA). Initially, radiological image data from patients with AA are collected as input. These data undergo preprocessing before being fed into a deep learning model for training. Upon completion of the training, the model is capable of producing various predictive outcomes, such as disease diagnosis, lesion segmentation, surgical assessment, prognosis, etc., offering references for doctors’ subsequent diagnosis and treatment decisions. Additionally, the figure outlines some of the current limitations in deep learning research in this context.

With the rapid advancement of computer hardware and deep learning theory, AI has found extensive application in the classification of medical image processing and is evolving swiftly (5). Currently, deep learning models have achieved diagnostic accuracy on par with radiologists for most tumor imaging, such as rectal cancer (6), breast cancer (7), lung cancer (8), and others. CNN and improved models are widely employed in medical image processing (9). In the field of vascular diseases, deep learning-based predictive models have yielded significant achievements in various areas, including coronary artery disease (10, 11), stroke (12, 13), and thrombotic disorders (14, 15). This study provides a summary of the current applications of deep learning technology in the diagnosis and treatment of AA disease. The workflow and application of deep learning research in radiological image data from patients with AA can be seen in Figure 1.

## 2 Application of deep learning in AA screening and diagnosis

AA is a life-threatening condition that can be effectively treated with surgical intervention if detected before rupture. The screening for AA is crucial for the prevention of this disease's progression. As AA are often asymptomatic in their early stages, screening is a key component in identifying potential risks. Traditional screening methods face challenges, including difficulty in detecting small or atypical aneurysms. Recently, deep learning technologies have shown great potential in enhancing the accuracy and efficiency of screening, particularly in image recognition and pattern analysis. These technologies not only improve diagnostic accuracy but also handle large volumes of data, playing a vital role in the early screening of AA. Deep learning technology can extract additional information from Non-Contrast CT (NCCT) scans that may be imperceptible to the human eye, facilitating AA diagnosis and assessment, with performance even comparable to CTA examinations. Golla, A. K. et al. (16) developed a diagnostic model that can automatically screen Abdominal Aortic Aneurysm (AAA) in NCCT scans and can be employed in a hospital environment. The study employed three different CNNs, including ResNet, VGG-16, and AlexNet, to analyze a dataset comprising 187 NCCT scans. ResNet addresses the vanishing gradient problem by introducing residual connections, allowing for the training of deeper networks; VGG-16 is characterized by its repetitive use of 3 × 3 convolutional layers and a deep stacked architecture, emphasizing the importance of network depth; AlexNet, as one of the earlier deep learning models, prevents overfitting through the use of ReLU activation functions and dropout techniques. Each model has its unique features and strengths, with ResNet being particularly effective in AAA screening tasks due to its deep network capabilities and residual learning mechanism. Among these models, ResNet outperformed the others. Its accuracy reached 0.856 and 0.953 in two separate validation sets, with the area under the receiver operating characteristic curves (AUROC) of 0.926 and 0.971. These results demonstrate the outstanding performance of the algorithm in AAA) screening and its potential for real-world medical application. However, its study still has shortcomings, firstly the relatively small size of the dataset may limit the generalization and robustness of the models. Additionally, while these models show excellence in AA screening, their capability in differentiating blood from other soft tissue components remains untested. This sets the stage for the study by Chandrashekar, A. et al. (17), they have proposed that sufficient information can be extracted from NCCT images to distinguish blood from other soft tissue components. The team developed software that generates CTA images from NCCT images, employing a deep learning algorithm based on Generative Adversarial Networks (GAN). GANs are a critical technology in the field of deep learning and have achieved remarkable success in image generation, style transfer, augmented reality, and various other applications. They included paired NCCT and CTA images from 75 AAA patients for training, comprising a total of 11,243 image pairs. Ultimately, both generated models were able to perform image translation tasks, with the Cycle Generative Adversarial Network model performing the best. It achieved an aneurysm cavity segmentation accuracy of 86.1% and a thrombus spatial morphology classification accuracy of 93.5%. This approach not only overcomes the limitations of data scale and model generalizability mentioned in Golla's study but also showcases the further application of deep learning technology in medical image transformation and more complex image processing tasks. Automatic AAA diagnosis on NCCT not only enables large-scale screening but also benefits patients who cannot undergo contrast-enhanced examinations, such as those with iodine allergies for whom contrast agents are contraindicated. This research provides a feasible alternative for patients who cannot undergo contrast-enhanced examinations and underscores the potential of deep learning technology in the future of medical applications.

Deep learning technology continues to excel in the screening and diagnosis of Thoracic Aortic Aneurysm (TAA). TAA is considered a risk factor for Acute Aortic Syndrome and must be accurately reported in every CT scan. An essential method for diagnosing TAA is to measure the diameter of the aorta. However, due to the complex anatomical structure of the thoracic aorta, TAA detection has remained challenging. To address this issue, Pradella et al. (18) utilized screening software AI-Rad (version 0.2.9.2, Siemens Healthineers, Forchheim, Germany), based on deep learning technology, to conduct TAA screening on a large population using non-CTA images. It was trained on more than 10,000 data sets for detection of aortic landmarks using deep reinforcement learning. Aortic segmentation was trained on more than 1,000 data sets using adversarial deep Image-to-Image network. According to the guidelines of the American Heart Association (AHA), this software measured the diameter of the thoracic aorta at nine different locations. The criteria for dilation were defined as a diameter exceeding 45 mm at the aortic sinus, sinus-aorta junction, ascending aorta, and near the aortic arch and exceeding 40 mm from the mid-arch to the descending aorta. This study successfully analyzed 18,243 CT cases, of which 12,092 were contrast enhanced CT (CECT) scans, and the remainder were NCCT scans. In the end, 97.0% of the cases (17,691 in total) were correctly classified, including 452 previously missed TAA cases. Compared to similar studies, AI-Rad employs deep reinforcement learning and adversarial deep Image-to-Image network for detection and segmentation tasks. Deep reinforcement learning combines the advantages of deep learning and reinforcement learning, while adversarial deep Image-to-Image network, through dual neural network adversarial training, enhance model accuracy and stability. Additionally, its training dataset originates from various manufacturers, granting the model superior generalization capabilities. AI-Rad can also generate 3D volumetric rendered images, allowing doctors to observe the measurements of the aorta more intuitively. (See Table 1).

**Table 1:** Summary of studies on the AA screening and diagnosis.

Author	Publication date	Research objectives	Imaging type	Patients	DL model	Predicted outcome accuracy
Golla, A. K. (16)	2021	Screening for AAA	NCCT	A dataset consisting of 187 heterogenous CT scans.	ResNet、VGG-16 和 AlexNet	In the first dataset it achieved an accuracy of 0.856 and AUC of 0.926. In second dataset it ran accuracy of 0.953 and AUC of 0.971
Chandrashekar, A. (17)	2023	Generation of CTA images using NCCT images	NCCT	Paired NCCT and CTA images of 75 AAA patients, totaling 11,243 image pairs	GAN	aneurysm lumen segmentation accuracy (Cycle-GAN: 86.1% ± 12.2% vs. Con-GAN: 85.7% ± 10.4%) and thrombus spatial morphology classification accuracy (Cycle-GAN: 93.5% vs. Con-GAN: 85.7%).
Pradella, M. (18)	2022	Screening for TAA	CECT、NCCT	18,243 CT scans (45.7% female) were successfully analyzed by AIRad	The DL-prototype (AIRad, Siemens Healthineers, Germany)	AIRad correctly assessed the presence or absence of TAA in 17,691 exams (97%), including 452 cases with previously missed TAA independent from contrast protocol.
Spinella, G. (19)	2023	Screening for AAA	CTA	73 thoraco-abdominal CTAs (48 AAA and 25 control CTA)	2.5D CNN	The pipeline correctly classified 47 AAA out of 48 and 24 control patients out of 25 with 97% accuracy, 98% sensitivity, and 96% specificity.

AA, aortic aneurysm; CNN, convolutional neural networks; CECT, contrast enhanced CT; NCCT, non-contrast enhanced CT; CTA, computed tomography angiography; AUC, area under the curve; GAN, generative adversarial network.

## 3 Application of deep learning in AA segmentation

Segmenting lesions in CT images of AA is crucial for guiding surgical decisions and subsequent treatment. However, existing segmentation methods are often time-consuming and challenging to apply in everyday clinical practice. To address this challenge, Siriapisith, T (20). introduced a deep learning-based novel AAA segmentation method, employing a CNN structure enhanced with coordinate information. This approach led to improved segmentation accuracy, achieving impressive Dice similarity coefficients (DSC) of 97.13% on CTA images and 96.74% on NCCT images. DSC, a statistical measure, quantifies the similarity between two sets, making it ideal for assessing segmentation accuracy in medical imaging. Additionally, they implemented transfer learning, a technique where a model developed for one task is reused on a second, related task, which here involved applying knowledge from preoperative datasets to EVAR postoperative datasets. This resulted in DSCs of 95.66% and 94.90% for postoperative aneurysm segmentation on CTA and NCCT datasets, respectively.

Mohammadi et al. (21) conducted a study with the aim of creating a fully automated model for abdominal region segmentation, AAA detection, and disease severity grading using CTA images. Their model consisted of three key steps: firstly (1), a CNN-based classifier was designed to categorize the abdomen into four distinct classes, including the abdominal inner region, aorta, body boundaries, and bones. Then (2), once the aorta was successfully detected, they used the Hough circle algorithm to precisely define its edges and measure its diameter. Finally (3), based on the detected aortic diameter, they categorized the disease into three risk levels: high, medium, and low. The model performed exceptionally well, achieving accuracy, precision, and sensitivity of 97.93%, 97.94%, and 97.93%, respectively. Additionally, a detection accuracy of 98.62% was achieved for the aortic region, and the Hough circle algorithm accurately classified 120 aortic regions with a precision of 98.33%. In summary, all steps of this classifier yielded the expected results. Another researcher, Abdolmanafi (22), utilized a Resnet-based fully convolutional network (FCN) with dilated convolutions as the deep learning architecture. They employed experienced vascular radiologists to manually delineate the contours of the aorta, the wall, and the intraluminal structures, and used the results of these expert delineations as the gold standard to train the deep learning model. The model includes three steps: First, it detects the “aorta” as a whole; second, it masks the original image to remove all surrounding similar organs and structures, thereby achieving more precise wall segmentation; finally, it detects the intraluminal structures and whether AAA is present. Notably, compared to expert manual segmentation, the results of automatic segmentation show good consistency, with a BF-Score (Boundary F1 Score) of 0.97 ± 0.03 and an IoU-Score(Intersection over Union) of 0.98 ± 0.02.

4D-flow technology, a non-invasive method based on MRI, measures blood flow using 3D images and time series data. It captures dynamic blood flow details like velocity, direction, and location in 3D space, aiding in the assessment of AA. Marin-Castrillon, D. M. et al. (23) successfully applied this technology for automated segmentation of the TAA region in 4D-flow MRI images. They used a U-Net based model, treating each image frame independently. The method achieved impressive accuracy, with a DSC of 0.90 and an average Hausdorff Distance (HD) of 9.58 mm, indicating its potential for wider use in TAA analysis.

Intraluminal thrombus is a significant factor in the progression of AA, and its presence is associated with the aneurysm sac and rupture. Precise quantification and volume analysis of thrombus are essential for better assessing the risk of AA rupture. Thrombus segmentation is a challenging task due to its irregular boundaries and the lack of clear definition resulting from adjacent structures with similar intensity values and low boundary contrast. Brutti, F (24). introduced a fully automated process for detecting and segmenting thrombus in AAA patients using CTA images, coupled with an analysis of AAA geometry. This approach creates polygonal models of the thrombus and intraluminal space, including the automated extraction of the intraluminal centerline to calculate aneurysm and intraluminal space diameters. The model's thrombus segmentation showed a DSC of 0.89 when compared to expert manual segmentation. Additionally, the AAA geometric analysis indicated a high intraclass correlation coefficient (ICC) of 0.92, with an average absolute diameter difference of 3.2 mm. ICC, a measure of reliability or agreement, indicates a strong concordance in measurements. These findings suggest that the developed deep learning model is effective in segmenting intraluminal thrombus in AAA patients. Lareyre, F (25)., integrated a feature-based expert system with deep learning algorithms to achieve a fully automatic segmentation of the abdominal vascular system. The results showed that this hybrid approach outperformed the expert system in intraluminal segmentation (volume similarity: 0.8128, DSC: 0.8266). Furthermore, the hybrid approach improved thrombus segmentation accuracy (volume similarity: 0.9404, DSC: 0.8918) compared to the expert system. (See Table 2).

**Table 2:** Summary of studies on the AA Segmentation.

Author	Publication date	Research objectives	Imaging type	Patients	DL model	Predicted outcome accuracy
Siriapisith, T. (20)	2022	Segmentation of AAA	CECT、NCCT	High resolution CECT and NCCT images containing 64 slices from each of 200 patients.	UNet、AG-DSV-UNet、VNet、ResNetMed 、DenseVoxNet	The best accuracies on NCCT and CECT images have average dice scores of 97.13% and 96.74%, respectively.
Mohammadi (21)	2019	Abdominal region segmentation, AAA detection, and disease severity classification	CTA、CECT	10 patients CT and CTA datasets	CNN	Abdominal inside region, aorta, body border, and bone with the accuracy, precision, and sensitivity of 97.93, 97.94, and 97.93% respectively.
Abdolmanafi (22)	2022	Segmentation of AAA	CTA	6,030 CT slices from abdominal CT scans obtained from 56 different patients with AAA.	FCN	The best model achieved 96.8640% accuracy (99.3794% sensitivity and 94.0271% specificity) in the validation set and 100% (case accuracy) and 93.3333% (image accuracy) in the test set.
Marin-Castrillon, D. M. (23)	2023	Segmentation of TAA	4D flow MRI	36 patients with TAA	U-Net	The segmentation performance was 0.90 ± 0.02 for the DSC and the mean HD was 9.58 ± 4.36 mm.
Brutti, F. (24)	2022	Segmentation of AAA thrombus	CTA	Dataset of 85 CTA scans	U-Net	The CNN-based classifier DSC of 0.89 is achieved. The AAA geometry analysis provided an ICC of 0.92.
Lareyre, F. (25)	2021	Segmentation of AAA thrombus	CTA	93 patients	U-Net	The hybrid approach demonstrated a good accuracy for lumen segmentation (volume similarity: 0.8128 and DSC: 0.8266).

AA, aortic aneurysm; CNN, convolutional neural networks; CECT, contrast enhanced computed tomography; NCCT, non-contrast enhanced CT; CTA, computed tomography angiography; AUC, area under the curve; MRI, magnetic resonance imaging; DSC, dice similarity coefficient; ICC; intraclass correlation coefficient.

## 4 Application of deep learning in AA surgery assistance

Surgery is a crucial treatment option for AA, and deep learning technology, through the evaluation of patients' preoperative, intraoperative, and postoperative imaging, can provide a more precise assessment of patients, assist in developing surgical plans, and evaluate postoperative complications. Measuring and assessing the aneurysm is the foundation of surgical decision-making for AA patients. However, manual measurements by clinical physicians are subject to errors and lack time repeatability, which may lead to delays in intervention or unnecessary surgeries. Bratt, A. et al. (26) trained a deep learning assessment model capable of automatically measuring aortic volume and diameter from preoperative CTA images. When compared to measurements by three radiologists, the deep learning model exhibited better time repeatability in volume (*p* < 0.008) and diameter (*p* < 1e-5). The repeatability metrics were comparable to the variability among manual assessors as reported in the past. Adam, C. et al. (27) used deep learning technology to detect and assess the maximum aortic diameter in AAA patients' preoperative and postoperative CTA images. The training dataset included 489 CTA images. The authors compared the maximum cross sectional diameter measurements manually conducted by two experienced aortic surgeons, three vascular surgery residents, and two general radiologists with the trained model. Ultimately, compared to expert measurements, the deep learning model exhibited a median absolute difference of 1.2 mm, outperforming the measurements of ordinary physicians. The manual measurements by clinical physicians may vary due to individual experience, differences in judgment standards, and the state at the time of evaluation. These factors, combined, inevitably lead to errors in measurements and lack of repeatability over time. In contrast, the deep learning model, by learning from a large dataset, provides a more consistent and objective measurement method, reducing the impact of human factors and thus enhancing the reliability of the measurements. Jiang et al. (28) introduced a novel method combining a computational model (vascular Growth and Remodeling, G&R model) with deep learning (Deep Belief Network, DBN) to address these challenges. The G&R model generates a limited simulated dataset, which, combined with patient follow-up data, is used to train the DBN. The model, tested with CT scan images from 20 patients, shows better performance in predicting AAA expansion compared to traditional models.

Patients with TAA undergoing Thoracic Endovascular Aortic Repair (TEVAR) require preoperative feasibility assessment and planning to determine adequate Landing Zones (LZs) for stent-graft deployment. Saitta, S. et al. (29) developed an automated technology based on CTA images for preoperative assessment in TEVAR. This system automatically segments the thoracic aorta, detects Proximal Landing Zones (PLZs), and quantifies essential geometric features such as curvature, diameter, and angles. Tested on 465 CT scans, the technique achieved a DSC of 0.95 for automatic segmentation and measurement, and was further validated in 9 additional patients, providing accurate surgical planning information for doctors. However, the study has certain limitations. Firstly, the technology relies on preoperative CTA images for planning, but actual surgery typically depends on Digital Subtraction Angiography (DSA) images, which may limit its practical utility in guiding operations. Further integration of DSA images could enhance its guidance value during actual procedures. Secondly, the technology focuses on planning for the thoracic aorta, and its applicability to AAA surgeries has not yet been explored. Kappe et al. (30) developed a fully automatic aortic stent-graft segmentation method based on DSA images acquired during EVAR surgery. They trained a 2D CNN with a U-Net architecture for stent-graft segmentation on DSA images. Through cross-validation, they obtained promising results with an average DSC of 0.957 and a median DSC of 0.968. Furthermore, the mean and median surface distance were 1.266 mm and 0.870 mm, respectively. This method provides robust assistance and assessment for aortic stent-graft implantation surgery, ensuring the accuracy of stent placement, visualizing endoleaks, and facilitating smooth operation of various functions such as image fusion correction.

Following EVAR for aortic intramural hematoma, long-term follow-up is essential to prevent life-threatening complications related to persistent type 2 endoleaks. Wang et al. (31) created a deep learning model to predict whether patients would experience severe adverse events associated with type 2 endoleaks. The training dataset included 10,240 CTA images from 75 patients with type 2 endoleaks, and the test set included 19 patients. In the test set, the deep learning model exhibited promising predictive performance, with an AUC of 0.917, accuracy of 0.842, and an F1 score of 0.897. However, a limitation of this study is that its training dataset is relatively small, including CTA images from only 75 patients. This may affect the model's general applicability and accuracy in a wider patient population. In response to this challenge, Hahn, S (32). collected postoperative CTA images (*N* = 334) from 191 patients to build a deep learning model for predicting postoperative type 2 endoleaks. The model also measured the diameter, area, and volume of the AA. The best type 2 endoleak detection model achieved a 0.94 AUROC and an optimized accuracy of 0.89 on a balanced dataset. The authors stated that further testing will be conducted on larger datasets. (See Table 3).

**Table 3:** Summary of studies on the AA surgery assistance.

Author	Publication date	Research objectives	Imaging type	Patients	DL model	Predicted outcome accuracy
Bratt, A. (26)	2021	Measures of volume and diameter	CTA	2,835 patients	U-Net	Deep learning models show better time repeatability for volume (*p* < 0.008) and diameter (*p* < 1e-5) measurements
Adam, C. (27)	2021	Detect and assess maximum aortic diameter	CTA	551 patients	CNN	The median absolute difference with respect to expert's measurements ranged from 1 mm to 2 mm among all annotators
Saitta, S. (29)	2022	Preoperative measurement of stent placement position	CTA	465 CT scans	U-Net	The trained CNN yielded a mean DSC of 0.95 and was able to generalize to 9 pathological cases of thoracic aortic aneurysm, providing accurate segmentations.
Kappe, K. O. (30)	2022	Fully automatic segmentation of the stent graft	DSA	DSAs of 47 AAA patients treated with EVAR	U-Net	An average DSC of 0.957 and median of 0.968. The mean and median of the average surface distance are 1.266 mm and 0.870 mm, respectively.
Wang, Y. (31)	2022	Predict the outcome of persistent type 2 endoleaks after EVAR	CTA	94 patients with persistent type 2 endoleaks	CNN	Achieved an AUC of 0.917, accuracy of 0.842, and F1 score of 0.897.
Hahn, S. (32)	2020	Endoleak detection and measurement of aneurysm diameter, area, and volume	CTA	191 unique patients undergoing EVAR	Retina Net、ResNet-50、U-Net	The best model of binary endoleak detection obtained an AUROC of 0.94 with an optimized accuracy of 0.89 on a balanced data set.
Jiang (28)	2020	Predicting AAA expansion and deciding when to perform surgery	CT	20 patients	Deep Belief Network (DBN)	DBN can predict the enlargements of AAAs with an average relative error of 3.1%, which outperforms the classical mixed-effect model by 65%.

AA, aortic aneurysm; CNN, convolutional neural networks; CECT, contrast enhanced CT; AUC, area under the curve; CTA, computed tomography angiography; DSC, dice similarity coefficient; EVAR, endovascular abdominal aortic aneurysm repair; DSA, digital subtraction angiography.

## 5 Application of deep learning in AA progression and prognosis prediction

Predicting the growth rate and pattern of AAA is crucial for early treatment and surgical intervention. Capturing key features, such as the accumulation of blood flow and intraluminal thrombus, plays a vital role in revealing the complex mechanisms underlying vascular adaptation, ultimately improving the ability to predict AAA growth. However, there is a high inter-patient variability in the local correlations between hemodynamic indices, biological characteristics, and morphological features with AAA growth rate, making biochemical and mechanical processes in time and space not fully understood. Rezaeitaleshmahalleh, M. et al. (33) used structural information of intraluminal thrombus to predict the growth status of AAA. They divided 54 AAA patients into two groups: slow growth (<5 mm/year) or rapid growth (≥5 mm/year). They generated 3D geometric AAA models using a deep learning image segmentation model and predicted the AAA's growth status through automated analysis. The prediction model had an AUROC of 0.89 and an overall accuracy of 83%. However, solely analyzing the structural information of thrombus to assess the prognosis of an AAA might result in an incomplete description of the complex biomechanical characteristics of AAA. Therefore, it is still necessary to collect and analyze multidimensional information of the localized lesion. Kim, S. et al. (34) employed a CNN approach to incorporate important multiphysical features related to the progression mechanism of AAA and validate their impact on AAA growth prediction. Testing four feature combinations from 54 patients, including radius, intraluminal thrombus thickness, time-averaged wall shear stress, and growth rate, the results showed that utilizing multiphysical features significantly improved AAA growth prediction, demonstrating that the proposed architecture surpasses previous state-of-the-art methods in this field. However, there are still shortcomings in this study. For example, studies mainly focus on the physical characteristics of AAA, ignoring the influence of biochemical indicators on prognosis. And while their predictive model performed statistically well, the general applicability of this approach across different patient populations remains to be validated. Therefore, future studies need to further explore the specific characteristics of different types and stages of AAA patients to improve the accuracy and generalization of predictive models.

EVAR for AAA has advantages such as minimal trauma, fast postoperative recovery, shorter hospital stays, and lower mortality and morbidity rates. However, long-term follow-up results show a higher risk of postoperative complications and reinterventions compared to open surgical repair. Therefore, guidelines recommend lifelong follow-up for patients undergoing EVAR (35, 36). Wang, Y (37). developed and compared multimodal models based on morphological features, deep learning, and radiomic features to predict the risk of EVAR-related Serious Adverse Events (SAEs) after EVAR. The results of the multimodal models showed that the radiomic model based on logistic regression had superior predictive performance (AUC 0.93, accuracy 0.86, F1 score 0.91) compared to the morphological feature model (AUC 0.62, accuracy 0.69, F1 score 0.81) and the deep learning model (AUC 0.82, accuracy 0.85, F1 score 0.89). Overall, all three models can assist in predicting the risk of SAEs relatively accurately. Caradu, C. et al. (38) evaluated a fully automated software named PRAEVAorta, which is designed based on the classic U-Net architecture. It aims to assess the development and associated risks of AAA after EVAR. By automatically analyzing post-EVAR CT images, it can measure the volume, surface, neck, and maximum diameter of AAA. The study results indicated that the measurements of the software were highly correlated with manual correction methods and were highly accurate. Moreover, the segmentation speed of the software is nine times faster than traditional manual correction methods, significantly improving clinical workflow efficiency. This technology could become a crucial adjunct for EVAR follow-up through the early detection of sac evolution, which might reduce the risk of secondary rupture. TAA is a disease that needs monitoring and treatment. With age, the aorta can dilate, become stiff, lose its elasticity, and may eventually rupture, leading to aortic dissection, which has a high mortality rate. The primary criterion for determining when a patient should undergo surgery is aortic diameter. However, it has been shown that aortic diameter alone is not sufficient to predict aortic dissection, indicating that other features should be considered. Markodimitrakis, E. et al. (39) aimed to assess the elastic properties of four different quadrants of the ascending aorta in TAA patients to evaluate a patient's aortic compliance and predict the risk of TAA developing into aortic dissection. They included 73 cine-MRI sequences of the ascending aorta and experimented with various deep learning architectures with different hyperparameters and settings to automatically segment the aortic contour on each image and then automatically calculate aortic compliance. This included U-Net and other modified architectures, such as Residual-U-Net (where each sub-module of U-Net is replaced with a residual connection and a dense layer), Attention-U-Net (which introduces an attention mechanism to adjust the encoder's output characteristics and provide the decoder with knowledge about high-level spatial information through attention gates), Attention-Residual-U-Net (inspired by the ResNet, implementing both residual blocks and attention gates), and Recurrent-Residual-U-Net (inspired by RNN, combining RNN and residual blocks). Among all models attempted, the U-Net network performed the best, with a DSC of 98.09% and a HD of 4.88 mm. The results showed that the lateral and posterior quadrants were stiffer, while the central and anterior quadrants exhibited the lowest aortic stiffness. The *in vivo* stiffness trends matched the values obtained ex vivo. The developed automatic segmentation method is robust, clinically compatible, and has reliable predictive capabilities. (See Table 4).

**Table 4:** Summary of studies on the AA progression and prognosis prediction.

Author	Publication date	Research objectives	Imaging type	Patients	DL model	Predicted outcome accuracy
Kim, S. (34)	2023	Prediction of AAA growth	CTA	54 patients	patch-based CNN	The results demonstrate the superiority of the presented architecture to previous state-of-the-art methods in AAA growth prediction.
Rezaeitaleshmahalleh, M. (33)	2023	predict AAA growth status	CTA	54 patients with intraluminal thrombus in their AAA	CACU-Net	Achieved an AUROC of 0.89 and a total accuracy of 83%.
Wang, Y. (37)	2022	Predicting outcomes after EVAR	CTA	979 patients underwent elective EVAR	DCNN	The logistics regression model had better predictive performance (AUC 0.93, accuracy 0.86, and F1 score 0.91)
Caradu, C. (38)	2022	EVAR surveillance	CTA	48 early post-EVAR CT scans and 101 follow-up CT scans	fully automated software (PRAEVAorta; Nurea, Bordeaux, France)	a mean DSC of 0.950, Jaccard index of 0.906, sensitivity of 0.929, specificity of 0.965, volumetric similarity of 0.973, and mean HD of 8.7 mm.
Markodimitrakis, E. (39)	2023	Predict aortic dissection in TAA patients	cine-MRI	73 patients	U-Net	Achieved a DSC of 98.09% and a HD of 4.88 mm.
Feng, H. (40)	2023	Automatic segmentation of thrombosed AD after surgery	CTA	167 patients with Stanford A AD	3D ResU-Net	0.903 in DSC, 0.828 in Jaccard index, and 2.209 in 95% HD

AA, aortic aneurysm; CNN, convolutional neural networks; CECT, contrast enhanced computed tomography; NCCT, non-contrast enhanced CT; AUC, area under the curve; MRI, magnetic resonance imaging; DSC, dice similarity coefficient; EVAR, endovascular abdominal aortic aneurysm repair; AD, aortic dissection; HD, hausdorff distance.

## 6 Discussion

Deep learning technology has now been applied to various aspects of the diagnosis and treatment of AA, playing a crucial role in timely disease detection and improving patient survival rates. However, as a category of disease research based on deep learning models, avoiding the shortcomings typical of conventional deep learning studies is challenging. These shortcomings are primarily reflected in several areas. For example, the size and quality of datasets directly impact the accuracy and generalizability of models. Currently, there is a significant lack of high-quality, large-scale, multi-center datasets in research. Additionally, the “black box” nature of deep learning models lacks the necessary transparency for diagnostic processes, which is unacceptable in clinical decision-making. The inadequacy of multi-center studies and coverage of diverse populations limits the universality and reliability of models. Moreover, clinical validation, model standardization, and legal and ethical considerations remain pressing issues in deep learning research. Lastly, the continuous updating and maintenance of models also face significant challenges with the rapid advancement of the medical field.

To address these limitations, several potential solutions can be explored. Firstly, establishing more collaborative projects and platforms that encourage different institutions to share and integrate data might help alleviate the lack of datasets. To increase model transparency and interpretability, researchers could explore new algorithms and techniques, such as explainable deep learning models, to provide clearer explanations of the decision-making process. Improving the universality and reliability of models could involve adopting cross-cultural and cross-geographical research designs to ensure models accurately reflect and cover the characteristics of diverse populations. Furthermore, working closely with regulatory bodies to establish standardized clinical validation processes could help ensure the safety and effectiveness of deep learning technologies. Lastly, developing adaptive learning systems that continually optimize and update models using new data could address the challenges posed by the rapid development of the medical field. Through these approaches, not only can the existing limitations be overcome, but the full potential of deep learning technology in clinical applications can also be harnessed, thereby enhancing the diagnosis and treatment of AA.

In many areas of AA diagnosis and treatment, deep learning technology is still lacking in research and requires more investment and attention. The following are some examples, which also represent key areas for future research:

**Lack of assessment of calcification:** The presence of aortic calcification has an impact on the development and rupture risk of AA, as it may lead to severe tissue overstretching in the surrounding areas. Higher aortic calcification scores are significantly associated with symptomatic and ruptured AA (41, 42). AI may provide an opportunity to develop software for rapid and objective quantification assessment of calcification in large patient datasets (43). However, there are still relatively few studies using deep learning techniques to assess calcification in AA patients.

**Lack of evaluation of stent placement during surgery:** EVAR and TEVAR are crucial methods for treating AA. The decision-making process regarding the size and shape of the stent, anchoring positions, and the extent of deployment significantly depends on the operator's personal experience and subjective assessment. Currently, deep learning research in the AA field primarily focuses on stent segmentation and measuring vascular diameters, with fewer studies addressing comprehensive judgment on various data or offering intuitive guidance to operators. Additionally, this technology largely relies on preoperative CTA images for surgical planning. Integrating DSA images into this technology could potentially enhance its guidance value during actual surgeries. Furthermore, while stent deployment is mainly utilized in planning for the thoracic aorta, its application in abdominal aortic aneurysm surgery planning requires further exploration and development.

**Lack of assessment of prognosis risk:** AA is a life-threatening disease, and real-time prognosis assessments for patients are crucial. In the field of non-deep learning artificial intelligence, there have been many achievements in the prognosis of AA patients, including the assessment of rupture risk (44, 45), in-hospital mortality risk (46, 47), 30-day mortality rate assessment (48), and more. Deep learning, compared to traditional machine learning algorithms, has higher fitting capabilities, and is expected to achieve better results in these areas.

AA due to its potential threat to life and the difficulty in diagnosis, has always been a focal point in medical research. Deep learning technology has provided us with a new and more accurate tool that can detect and locate AA from complex medical images with precision, thus offering the possibility for early intervention and treatment. Especially in the analysis of imaging and segmentation of the aneurysm, deep learning models have demonstrated significant advantages over traditional methods. More importantly, as the technology further develops, we expect deep learning to gain a deeper understanding of the pathogenesis of AA, providing more personalized and precise suggestions for treatment strategies. Additionally, with the deepening of interdisciplinary collaboration, we hope to discover more biomarkers and risk factors related to AA, thereby further enriching our understanding of this disease. Overall, deep learning technology has opened new doors for the diagnosis and treatment of AA, bringing endless possibilities for future medical research.

## 7 Conclusion

In this article, we delved deeply into the application of deep learning in the diagnosis and treatment of AA, showcasing the latest advancements in the field. We discovered that deep learning technology has not only made significant progress in enhancing the accuracy and efficiency of AA imaging but also demonstrated substantial applicability in precise treatment planning and risk assessment. Particularly in refining the planning of surgical interventions and in predicting the risk of aneurysm rupture, deep learning has shown a broad development perspective. The core contribution of this paper lies in synthesizing multiple key research outcomes and pointing out the gaps and future directions in current research. Our review provides a valuable source of information for researchers and clinicians in the field, emphasizing the crucial role and extensive development potential of deep learning in improving AA management. It lays the groundwork for further exploration in future studies related to this subject.
